# A potential cost savings analysis of a penicillin de-labeling program

**DOI:** 10.3389/falgy.2023.1101321

**Published:** 2023-03-30

**Authors:** Yilu Dong, Tracy N. Zembles, Mark Nimmer, David C. Brousseau, David Vyles

**Affiliations:** ^1^ Pediatric Emergency Medicine, Medical College of Wisconsin, Milwaukee, WI, United States; ^2^ Department of Enterprise Safety, Children's Wisconsin, Milwaukee, WI, United States

**Keywords:** allergy, penicillin allergy, allergy de-labeling, cost savings, drug prescriptions

## Abstract

**Introduction:**

Over 95% of patients documented as penicillin allergic can tolerate a penicillin without a reaction. Inaccurate documentation of penicillin allergy leads to more expensive alternative antibiotic prescriptions and an increased incidence of resistant infections.

**Objective:**

To understand the potential drug cost savings of a penicillin de-labeling program to a healthcare system.

**Methods:**

We evaluated patient visits with documented penicillin allergy who presented to the pediatric Emergency Department (PED) and 22 associated primary care clinics. Patients were included if they were discharged home with a non-penicillin antibiotic when the first-line treatment for the diagnosis would have been a penicillin. The potential cost savings were the sum of all visit-level cost differences between the non-penicillin prescription(s) and a counterfactual penicillin prescription. To factor in a 95% successful patient de-labeling rate, we repeatedly sampled 95% from the patients with the eligible visits 10,000 times to produce an estimate of the potential cost savings.

**Results:**

Over the 8-year period, 2,034 visits by 1,537 patients to the PED and 12,349 visits by 6,073 patients to primary care clinics satisfied eligibility criteria. If 95% of the patients could have been successfully de-labeled, it would have generated a cost saving of $618,653 (95% CI $618,617—$618,689) for all the corresponding payers in the system.

**Conclusions:**

Implementing a penicillin de-labeling program across a healthcare system PED and its associated primary care clinics would bring significant cost savings. Healthcare systems should rigorously evaluate optimal methods to de-label patients with reported penicillin allergy.

## Introduction

Penicillin (PCN) allergy is often documented in patient records. Approximately 10% of the United States population and 8%–25% of most populations studied globally are labeled as PCN allergic; however, most diagnoses of PCN allergy are made in childhood and relate to events that are either not allergic in nature, are low risk for immediate hypersensitivity, or are a potential true allergy that wanes over time (1, 2). Studies show that most patients with a reported allergy to PCN can tolerate a PCN antibiotic without developing severe reactions (1, 3–6). Inaccurate documentation of PCN allergy leads to the use of less effective, more toxic and expensive alternative prescriptions of antibiotics with inappropriately broader coverage than PCN, placing patients at higher risk of antimicrobial resistance, treatment failure, longer hospital stays, and even increased mortality (2, 4, 6–9). To understand the potential value (reduction in quality-adjusted cost) a PCN de-labeling program can bring to a healthcare system, this study takes the first step to examine the potential cost savings a PCN de-labeling program could have brought to a large children's care network over the past 8 years.

## Method

**Figure 1:**
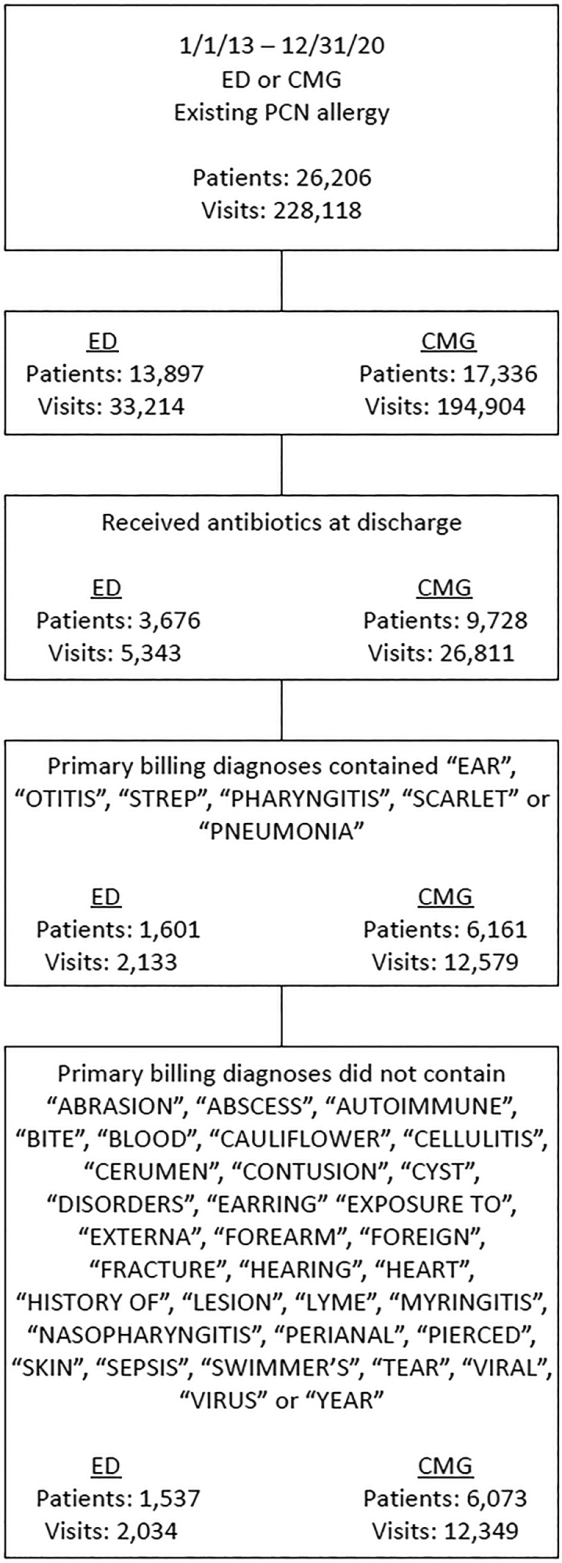
Eligibility criteria to consider PCN as the preferred antibiotics after de-labeling. ED, Emergency Department; CMG, Children's Medical Group (primary care); PCN, Penicillin.

The study was performed at Children's Wisconsin, a comprehensive pediatric care center affiliated with the Medical College of Wisconsin, located in Milwaukee, Wisconsin. We evaluated patient visits with documented PCN allergy who presented to the pediatric emergency department (PED) and 22 associated primary care clinics (children's medical group, CMG) from 01/01/2013 to 12/31/2020. Visits were included in the analysis if patients had a PCN allergy and were discharged with an antibiotic prescription. Based on the primary billing diagnoses of the visits, we systematically applied our eligibility criteria (Figure 1, for which we use PCN as first-line antibiotics^1^) to identify visits that we would have preferably been prescribed a PCN antibiotic instead of a non-PCN antibiotic if the patients had been de-labeled (hereinafter these visits are referred to as the “eligible visits”).

Among all the eligible visits, visit-level cost savings were calculated as the difference between the total cost of the actual antibiotics prescribed for the visit and the optimal cost, which equaled the cost of a single PCN prescription. For a small percentage of visits (0.6%) with multiple non-PCN antibiotics prescriptions while PCN was preferred under the eligibility criteria, since the infections of the outpatient populations were generally uncomplicated, we assumed that a single PCN prescription could be sufficient to substitute the multiple non-PCN antibiotics prescribed to facilitate the large-scale review of visits in the past eight years. The total cost savings were simple summations of all the visit-level cost savings within the PED or the CMG. Conformance to PCN allergy labels was defined as if a provider prescribed non-PCN antibiotics when a patient was labeled as PCN allergic.

Nine types of antibiotics covered in the study were: first, second and third generation cephalosporins, fluoroquinolones, lincosamides, macrolides, tetracyclines, sulfa drugs, and PCN (amoxicillin). The average per-visit prescription cost was calculated based on the average cash price (without coupon) from the website www.GoodRx.com, in a capsule formulation with a prescription duration of 7 days (Table 1) (10).

**Table 1:** Potential drug cost savings by types of antibiotics.

Types of Antibiotics	Average per-visit Prescription Cost^a^	Emergency Department			Children's Medical Group		
		Actual Prescriptions Filled	Penicillin Preferred if De-labeled	Potential Cost Savings^b^	Total Prescriptions Filled (actual)	Penicillin Preferred if De-labeling	Potential Cost Savings^b^
First gen. cephalosporin (Keflex/Cephalexin)	$ 24.94	368	60	$ 775.20	1,356	433	$ 5,594.36
Second gen. cephalosporin (Cefuroxime)	$ 80.32	62	35	$ 2,390.50	324	129	$ 8,810.70
Third gen. cephalosporin (Cefdinir)	$ 76.65	1,940	1,155	$ 74,647.65	12,497	6,836	$ 441,810.68
Fluoroquinolones (Ciprofloxacin)	$ 30.14	217	16	$ 289.92	136	23	$ 416.76
Lincosamides (Clindamycin)	$ 77.92	1,065	143	$ 9,423.70	981	193	$ 12,718.70
Macrolides (Azithromycin)	$ 31.81	1,012	508	$ 10,053.32	9,131	4,180	$ 82,722.20
Tetracyclines (Doxycycline)	$ 74.34	88	5	$ 296.02	636	26	$ 1,620.32
Sulfa drugs (Bactrim)	$ 15.77	384	16	$ 60.00	916	138	$ 517.50
Penicillin (Amoxicillin)	$ 12.02	363		$ -	898		$ -
Total Cost Savings ^c^				$ 97,307.45			$ 553,875.34

^a^ Calculation based on the average cash prices (without coupon) extracted from the website www.GoodRx.com, accessed on Dec. 15th, 2021, in a capsule formulation with a prescription duration of 7 days.

^b^ Formula for Potential Cost Savings = Difference in average per-visit prescription cost * number of PCN prescriptions preferred if de-labeled. E.g., for first gen Cephalosporin: ($24.94—$12.02) * 60 = $775.20.

^c^ When transforming the original visit-level analysis to a summary table by types of antibiotics, the formula above for potential cost savings will slightly inflate PCN prescriptions needed for visits with multiple antibiotics prescriptions (less than 1% of the visits had more than 1 antibiotic prescription). The total cost savings shown have adjusted for such inflations to be accurate.

A previous systematic review of publications on PCN allergy testing in the past 20 years found that 95.1% [95% confidence interval (CI) 93.8%–96.1%] of the populations, consistently in outpatient, perioperative, and inpatient settings, could pass a PCN skin test (4). Based on the meta-analysis, we also factored in a 95% successful de-labeling rate in our estimation by random sampling. Specifically, 95% of the patients were randomly sampled from the eligible patient pool and the total cost savings of their corresponding eligible visits were computed. We repeated this step 10,000 times, calculated the mean of the 10,000 estimates and provided its 95% CI. Using this resampling method, the classical central limit theorem (CLM) warranted the sample mean of the total cost savings would approximate a normal distribution and converge to the population mean (11). The simulation process was conducted in Stata version 17 (StataCorp LLC, College Station, Texas, United States). The Children's Wisconsin institutional review board (IRB) determined this project to be non-human subject research.

## Results

There was a total of 2,790,713 visits across the PED (517,874) and CMG (2,272,839) over the study period, among which, 228,118 visits (26,206 unique patients) had PCN allergy labels, leading to a prevalence rate of 8.2% at our institution. Of 33,214 PED visits (13,897 patients), 5,343 (16.1%) were prescribed antibiotics at discharge. The mean (SD) age was 7.7 (5.3) years, 2,729 (51.1%) were male, 4,231 (79.2%) were White, 589 (11%) were Black patients. In the PED, 2,034 visits (1,537 patients) satisfied the eligibility criteria to favor PCN prescriptions over the actual non-PCN prescriptions. The potential total drug cost savings were $97,307, with their breakdown by types of antibiotics in Table 1. If 95% of these patients could have been successfully de-labeled under a PCN de-labeling program, it would have generated a cost saving of $92,446 (95% CI $92,439—$92,454). Likewise, of 194,904 CMG visits (17,336 patients), 26,811 (13.8%) were prescribed antibiotics at discharge. The mean (SD) age was 7.6 (5.2) years, 13,390 (50%) were male, 21,546 (80.4%) were White, 2,588 (9.7%) were Black individuals. In CMG, 12,349 visits (6,073 patients) met the eligibility criteria to favor PCN prescriptions. The total potential drug cost savings were $553,875, and their breakdown by types of antibiotics was provided in Table 1. With a 95% successful patient de-labeling rate, there would have been a cost saving of $526,207 (95% CI $526,178—$526,235). The total cost savings across the sites would have been $618,653 (95% CI $618,617—$618,689) to all the corresponding payers in the healthcare system. Providers' conformance rates to patients' PCN-allergy labels were 93.2% and 96.7% at PED and CMG, respectively.

## Discussion

The study suggests a significant amount of potential drug cost savings a PCN de-labeling program could bring to patients and payers in a healthcare system. We also find that in patients with reported PCN allergy, most providers tend to prescribe PCN alternatives when the patient conditions call for it as the first choice. However, given a majority of the population labeled as PCN allergic can tolerate the medication without an allergic reaction, it would be more effective and less costly to prescribe it compared to non-PCN alternatives (1, 2). The change in prescription pattern brought about through a PCN de-labeling program would result in net cost savings to all payers in the health care system, and the higher provider's conformance rate to the PCN-allergy label, the greater potential values that a PCN de-labeling program could add.

There are important limitations to the study. First, as a retrospective study from a single medical center, our estimated potential cost savings may not generalize to other centers, where characteristics of the patient populations could be different, and this study does not seek to predict cost savings for other patient populations. Second, for the sake of review efficiency, the application of the eligibility criteria based on a keyword-filtering method, as well as only on the primary billing diagnoses of the visits may not present a full and accurate picture of patient conditions for every encounter, affecting the accuracy of our cost-saving estimations. Third, this study only focused on outpatient prescription patterns, leading to more conservative estimates of the cost savings. If inpatients were included, the potential cost savings could be even greater. Finally, this study focused on potential drug cost savings without factoring in operation costs of managing a PCN de-labeling program. Future studies incorporating such operation costs and its long-term quality improvements on patients (e.g., long-term adverse outcomes avoided and thus quality of life improved by switching from the suboptimal non-PCN antibiotics to PCN) could reveal a more comprehensive value picture that a PCN de-labeling program can bring to our patients.

In conclusion, implementing a penicillin de-labeling program would generate significant net drug cost savings to the payers. Healthcare systems should rigorously evaluate optimal methods to de-label patients with reported penicillin allergy.

## Data Availability

The raw data supporting the conclusions of this article will be made available by the authors, without undue reservation.

## Footnotes

^1^We do not treat urinary tract infections or skin infections with PCN at our institution. Outside of pneumonia we typically do not treat other respiratory infections with antibiotics at our institution as well.
